# Fibroblast growth factor 8: Multifaceted role in development and developmental disorder

**DOI:** 10.1016/j.gendis.2025.101524

**Published:** 2025-01-10

**Authors:** Huamin Yin, Lian Duan, Zhendong Wang, Li Liu, Jingling Shen

**Affiliations:** a Institute of Life Sciences, College of Life and Environmental Sciences, Wenzhou University, Wenzhou, Zhejiang 325035, China; b Central Laboratory, The First Affiliated Hospital of Wenzhou Medical University, Wenzhou, Zhejiang 325000, China; c Key Laboratory of Interventional Pulmonology of Zhejiang Province, The First Affiliated Hospital of Wenzhou Medical University, Wenzhou, Zhejiang 325000, China

**Keywords:** Developmental disorders, Embryonic development, FGF8, Organogenesis, Single cell RNA sequencing

## Abstract

Fibroblast growth factor 8 (FGF8), a secreted signaling molecule, involves in regulating cell survival, proliferation, migration, and differentiation. It exhibits a highly dynamic gene expression pattern throughout embryonic development, participates in craniofacial structures, limbs, internal organs, brain development, and is crucial during organogenesis. The dysregulation of precise localization and dosage of FGF8 at distinct embryonic stages can lead to developmental multiorgan abnormalities. This comprehensive review explores the *FGF8* expression in humans and mice, summarizes the involvement of FGF8 in various tissues including craniofacial, limbs, cardiovascular and urogenital system, nephrogenesis, lung, and brain development as well as developmental abnormalities resulting from the aberrant regulations of FGF8 such as skeletal abnormalities, ciliopathies, and holoprosencephaly.

## Introduction

The fibroblast growth factors (FGFs) family, comprising 22 multifaceted proteins, plays critical roles in normal development. Based on their encoded proteins, these factors are classified into three main categories: five subfamilies of paracrine FGFs (FGF1/4/7/8/9 subfamily), one subfamily of intracellular FGFs (FGF11 subfamily), and one subfamily of endocrine FGFs (FGF15/19 subfamily).^1^

The FGF8 subfamily is comprised of FGF8, FGF17, and FGF18. Initially identified in the mouse mammary carcinoma cell line (SC-3), FGF8 was originally named androgen-induced FGF-like growth factor (AIGF).^2^ The human *FGF8* gene is located on chromosome 10 (10q24.32).^3^ Alternative splicing of *FGF8* generates four isoforms, *FGF8a*, *FGF8b*, *FGF8e*, and *FGF8h*.^4^ Among them, *FGF8b* is the predominant expression isoform, with a stronger receptor-binding capability than other isoforms,^5^^,^^6^ and FGF8 protein contains 233 amino acids. In the mouse, the *Fgf8* gene resides on chromosome 19^7^ and produces eight potential protein isoforms, denoted as *Fgf8a* to *Fgf8h*. *Fgf8a*, *Fgf8b*, *Fgf8e,* and *Fgf8f* correspond to transcripts produced by human *FGF8* gene. The amino acid sequences between human and mouse are highly conserved, with 98%–100% sequence similarity.^4^ This conservation underscores the significance of FGF8 in evolutionary processes across species.

Members of FGF8 subfamily exhibit a classical N-terminal signal peptide and are known to activate IIIc splice forms of FGFRs 1–3 and FGFR4.^8^^,^^9^ As a crucial player in developmental biology, FGF8 holds a central position in signaling pathways such as MAPK/ERK, PI3K-AKT, and PLCγ/Ca^2+^, orchestrating various cellular processes including cell survival, proliferation, migration, and differentiation.^10^ Its integral roles in the intricate processes of mammalian growth and development are extensively documented in the scientific literature.^11^^,^^12^

## Expression profile of human and mouse FGF8

**Figure 1:**
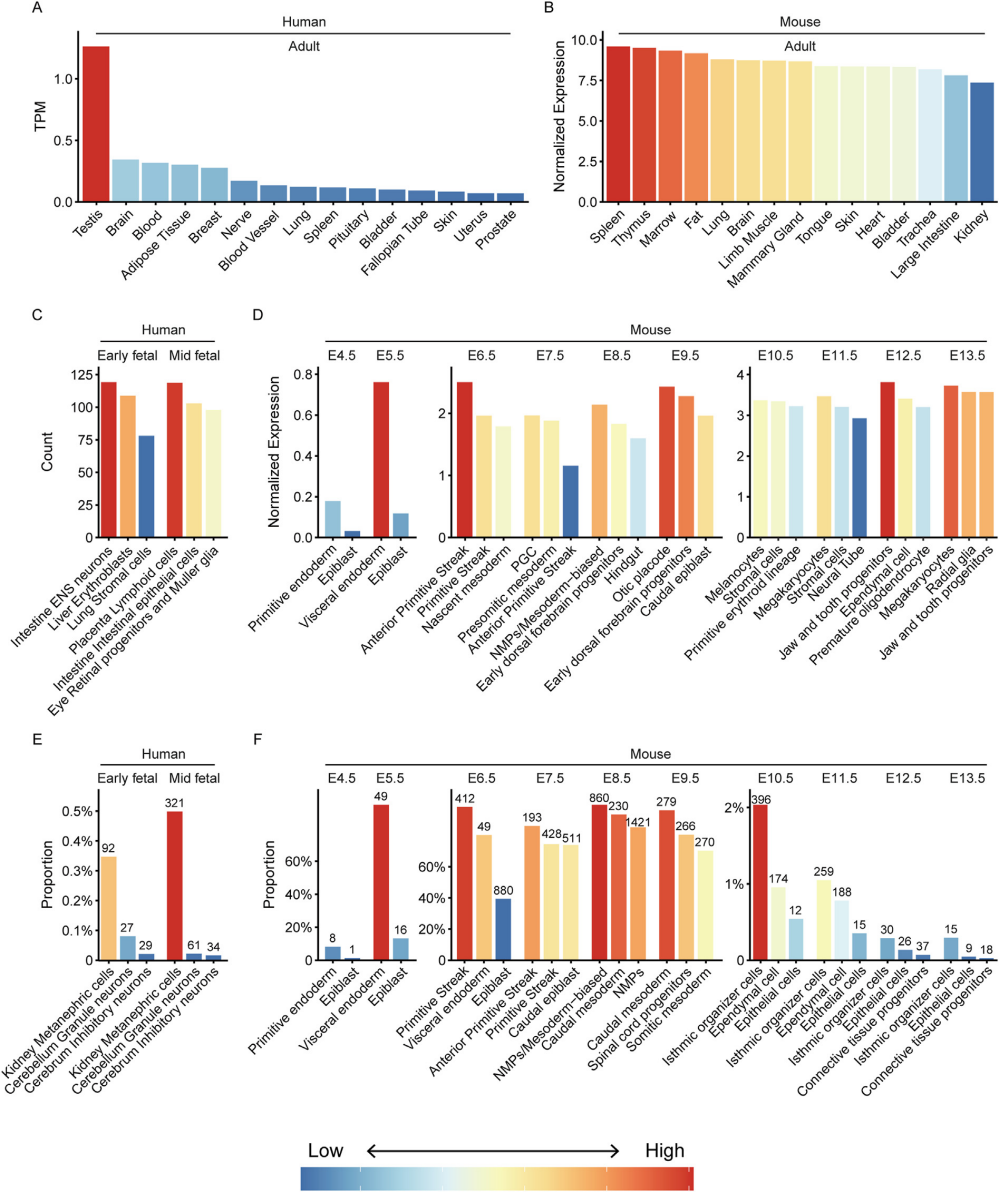
The human and mouse expression profile of FGF8. **(A)** Relative abundance of FGF8 in adult human tissues from the Genotype-Tissue Expression (GTEx V8) Project Consortium (TPM, transcripts per million reads). Data is available at https://www.gtexportal.org/home/datasets. **(B)** Relative abundance of Fgf8 in adult mouse tissues from Tabula Muris Compendium (https://tabula-muris.ds.czbiohub.org/). **(C)** FGF8 expression in fetal human, derived from single cell transcriptome analysis (https://cells.ucsc.edu/?ds=fetal-gene-atlas/). Top 3 celltypes with highest FGF8 expression per stage are displayed. **(D)** Fgf8 expression in mouse embryo (E4.5–13.5) from multi single cell transcriptome datasets (E4.5–5.5: ftp://ftp.ebi.ac.uk/pub/databases/scnmt_gastrulation; E6.5–9.5: https://cells.ucsc.edu/?ds = ext-mouse-atlas; E9.5–13.5: https://ftp.ncbi.nlm.nih.gov/geo/series/GSE119nnn/GSE119945/suppl/). **(E)** The proportion of FGF8-positive cell in fetal human. Top 3 cell types with highest FGF8 positive proportion per stage are exhibited. The numbers on bars represent the count of FGF8-positive cells. **(F)** FGF8 positive cell proportion in mouse embryo. PGC: Primordial germ cells; NMPs: Neuromesodermal progenitors.

Previous studies have consistently demonstrated the selective expression of *Fgf8* in various adult organs and tissues. For example, low-level expression of *FGF8* has been identified in organs such as the kidney, prostate, testis, breast and others, as reported in previous studies.^13^, ^14^, ^15^ This observation is supported by an extensive analysis of 17,382 RNA-Seq samples derived from 54 tissues across approximately 948 individuals in the Genotype-Tissue Expression (GTEx V8) Project Consortium,^16^ as depicted in Figure 1A. Furthermore, the *Fgf8* expression also has been detected in adult mouse testis from the fetal days 15–17.^17^ The comprehensive organizational level analysis of *Fgf8* expression from the Tabula Muris project, which integrates single-cell mouse transcriptome data from 20 organ tissues over 100,000 cells,^18^ reveals the similarity between the top 15 *Fgf8* expression levels in mice and humans. Notably, the spleen and thymus exhibit the highest expression levels in both species (Fig. 1B).

While *FGF8* is widely expressed during embryonic development, a comprehensive assessment of its expression in fetal development remains to be delineated. Single-cell transcriptome sequencing provides an avenue to explore the dynamic expression of *FGF8* at the cellular level. The latest data on human and mouse embryonic development were sourced from UCSC Cell Browser^19^ and PubMed. The human fetal gene expression profile encompasses about 4 million cells from 15 organs, collected between gestation days 72 and 129 and categorized into early fetal (<13 weeks) and mid fetal (>13 weeks).^20^ *FGF8* exhibits abundant expression in the early fetal stage in the enteric nervous system (ENS) neurons, liver erythroblasts and lung stromal cells. In the mid fetal stage, high expression is observed in placenta lymphoid cells, intestinal epithelial cells and retinal progenitors and Muller glia of eyes. Notably, the proportion of *FGF8*-positive cells remains consistent in both early- and mid-fetal, with the highest proportion in the kidney metanephric cells and neurons, indicating a continuous *FGF8* expression during development (Fig. 1C, E).

Integration of multiple datasets from mouse fetal development spanning embryonic days (E) 4.5 to E13.5^21^, ^22^, ^23^, ^24^ reveals highly dynamic *Fgf8* expression across various developmental stages, affirming its positive role in organogenesis (Fig. 1D, F). *Fgf8* expression is relatively high in the endoderm and epiblast at E4.5 and E5.5, shifting to the primitive streak from E6.5, and detected in primordial germ cells (PGC) at E7.5. Starting from E8.5, *Fgf8* expression is initiated in the brain and multiple organs. The proportion of *Fgf8*-positive cells consistently increases from E4.5 to E6.5, notably high in the visceral endoderm on E5.5 and the anterior primitive streak on E6.5. However, from E10.5, a low proportion of *Fgf8*-expressing cells is observed. Single-cell transcriptional profiling of human and mouse confirms the highly dynamic expression pattern of *FGF8* during embryonic development.

## FGF8 in craniofacial development and malformations

### FGF8 in craniofacial development

Craniofacial development commences around the 4th week in the human embryo with the formation of five distinct facial prominences in the pharyngeal arches through the differentiation of cranial neural crest cells (NCCs) into chondroblasts. These prominences give rise to various facial components, including the mandible, maxilla, palate, lips, and nose.^25^ Cranial NCCs, originating from the embryonic diencephalon, midbrain, and hindbrain, participate in the craniofacial development and regeneration.^17^ FGF8 emerges as a critical factor in guiding cranial NCC migration, differentiation, and survival.^26^ Notably, in *Wnt1-cre;Rosa26R-Fgf8* mice, augmented FGF8 signaling is found to maintain the progenitor status and multipotency of pre-migratory cranial NCCs-derived mesenchymal cells. In vitro experiments further elucidate that FGF8 signaling strongly induces adipogenesis while concurrently inhibiting osteogenesis in cranial NCC-derived mesenchymal cells, achieved through the antagonism or inhibition of BMP signaling.^27^ (Table 1).

**Table 1:** Putative FGF8 mutations and associated phenotypes.

Variant effect		Type	Inheritance	Genotype	Phenotype	Reference
DNA	Protein					
c.77C > T	p.Pro26Leu	Missenses	Dominant	HET	KS	5
c.379C > G	p.Arg127Gly	Missenses	Dominant	HET	KS	5
c.40C > A	p.His14Asn	Missenses	Unknown	HET	nIHH	5
c.118T > C	p.Phe40Leu	Missenses	Recessive	HOMO	nIHH	5
c.298A > G	p.Lys100Glu	Missenses	De novo	HET	nIHH	5
c.686C > T	p.Thr229Met	Missenses	Unknown	HET	AHH	5
	p.Asp73His	Missenses	De novo	Unknown	Bilateral cleft lip and palate	41
c.599A > G	p.Glu200Gly	Missenses	Dominant	HET	NSCLP	42
10q24.32		Duplication	De novo	Unknown	Congenital limb malformations	66
c.86_103dupGCCCTGCGCTGGGCAGGG	p.Gly29_Arg34dup	Duplication	Recessive	HET	VATER/VACTERL	108
c.77C > T	p.Pro26Leu	Missenses	Recessive	HET	VATER/VACTERL-like phenotype	108
c.763C > T	p.Arg127∗	Missenses	Dominant	HET	KS	141
c.769C > T	p.Arg129∗	Missenses	Recessive	HET	nIHH, severe GnRH deficiency.	141
c.566G > A	p.Arg189His	Missenses	Recessive	HOMO	HPE, microcephaly, micrognathia	144
c.646C > G	p.Gln216Glu	Missenses	Recessive	HET	SOD, agenesis of corpus callosum	144
c.356C > T	p.Thr119Met	Missenses	Recessive	HET	Semilobar HPE	146
c.317C > A	p.Ala106Glu	Missenses	De novo	HET	Semilobar HPE	146
c.385C > T	Arg129∗	Missenses	Recessive	HET	Alobar HPE, syntelencephaly	146
c.617G > A	p.Arg206Gln	Missenses	Dominant	HET	Microform, bilateral cleft lip and palate	146
c.118T > C	p.Phe40Leu	Missenses	Unknown	HOMO	KS	163
c.68A > T	p.Gln23Leu	Missenses	Unknown	HET	KS	163
c.298A > G	p.Lys100Glu	Missenses	Unknown	HET	nIHH	163
c.40C > A	p.His14Asn	Missenses	Unknown	HET	nIHH	163
c.77C > T	p.Pro26Leu	Missenses	Unknown	HET	nIHH	163
c.216G > A	p.Thr72Thr, splice defect	Synonymous	Unknown	Unknown	CPHD, delayed puberty	164
c.686C > T	p.Thr229Met	Missenses	Dominant	HET	Holoprosencephaly, microcephaly, seizures, cleft palate, severe neurological impairment	165

Abbreviations: HET, heterozygous; HOMO, homozygous; KS, kallmann syndrome; nIHH, normosmic idiopathic hypogonadotropic hypogonadism; AHH, adult-onset hypogonadotropic hypogonadism; NSCLP, nonsyndromic cleft lip and palate; GnRH, Gonadotropin-releasing hormone; SOD, septo-optic dysplasia; HPE, holoprosencephaly; CPHD, combined pituitary hor-mone deficiency.

FGF8 also plays a significant role in determining osteogenic cell fate within the hard palate. In *Shox2-cre;Rosa26R-Fgf8* mice, localized *FGF8* activation prompts the loss of the maxilla palatal process and leads to ectopic cartilage tissue formation. This enhanced FGF8 signaling acts as a negative regulator during osteogenic differentiation, exerting a synergistic impact on craniofacial osteogenesis in collaboration with Shh.^28^^,^^29^ Moreover, FGF8 participates in supporting the survival of mesenchymal cells in the first pharyngeal arch (PA1), crucial for the development of craniofacial skeletal elements.

FGF8 signaling is instrumental in establishing the head-caudal polarity of the PA1.^30^ On embryonic day E11.5 in mice, *Fgf8* expression is identified in molar epithelial progenitor cells, contributing to the formation of epithelial cells essential for tooth development.^31^ Abundant *Fgf8* expression persists in jaw and tooth progenitors at E12.5 and E13.5 (Fig. 1D). In summary, FGF8 emerges as a crucial role in cranial neural crest cell migration, differentiation, and survival, exerting influence on mesenchymal cell fate, osteogenesis, and contributing to the variation in disease severity within the lower jaw, specifically Meckel's cartilage, with directional asymmetry.

### FGF8 dysregulation results in craniofacial malformations

Craniofacial development is an intricately regulated process susceptible to perturbations arising from genetic and environmental factors, resulting in diverse developmental anomalies. Frequently, these anomalies can be traced back to disruptions in the formation, survival, migration, or differentiation of cranial NCCs. Dysregulation of *Fgf8* expression directly impacts the migration, proliferation and survival of cranial NCCs, thereby contributing to abnormalities in craniofacial development.^25^^,^^27^^,^^32^ The specific inactivation of FGF8 signaling in PA1 ectoderm in *Fgf8;Nes-cre* mice leads to first arch syndromes, similar to mandibulofacial dysostosis.^33^ Reduced *Fgf8* expression has been associated with disruptions in pharyngeal arch development.^34^^,^^35^ Its inhibition, along with *Bmp7* and *Shh*, has been shown to affect the second arch development.^36^ Disease severity variation is associated with Fgf8 dosage, particularly in the development of the lower jaw, including Meckel's cartilage and its derivatives, exhibiting directional asymmetry with the left side more affected than the right^37^

### Mandibulofacial abnormalities

Micrognathia is a serious congenital anomaly characterized by a narrowed or shortened mandible. Clinically, newborns with such jaw deformities frequently experience breathing difficulties and feeding intolerance. More critically, complications like glossoptosis or retroglossia can result in Pierre Robin sequence (PRS). In PRS, the non-descending tongue obstructs the elevation of the palatal framework, leading to the development of a cleft palate. The ectopic activation of *Fgf8* in mesenchyme is associated with micrognathia. This anomalous activation induces regression in masseter, disrupts the patterning and differentiation of masseter tendon, and diminishes the mechanical force transmitted to the mandibular bone, collectively contributing to the manifestation of micrognathia.^38^ This study provides a novel mechanism for the pathogenesis of micrognathia, activating *Fgf8* disrupts tenogenesis lead to impaired osteogenic specification rather than differentiation in periosteal progenitors. Genetic interplay between *Fgf8* and *Foxc1* in mammalian jaw patterning, coupled with observed dosage-dependent variations in syngnathia severity within *Fgf8*^*Null/+*^*;Foxc1*^*−/−*^ mice, highlights the intricate and multifaceted role of *Fgf8* in craniofacial development.^37^^,^^39^ Disruptions in *BMP4* and *FGF8* gene regulatory networks in NCCs proximal to the maxillomandibular junction, resulting from *Gata3* disruption, lead to craniofacial microsomia and syngnathia in mice (Table 2).^40^ Notably, *Fgf8*^*H/-*^ hypomorphic embryos exhibit craniofacial abnormalities reminiscent of human 22q11 syndromes, featuring micrognathia and bony cleft palate.^32^

**Table 2:** Phenotypes of Fgf8 mouse models.

Genotype	Effect on FGF8 function	Additional genes	Phenotype	Reference
*Wnt1*^*Cre*^*;Rosa26R*^*Fgf8*^	GOF		Exencephaly, absent eye, absent ear auricles	27
			PTA	94
*Shox2*^*Cre*^*;Rosa26R*^*Fgf8*^	GOF		Absent palatine process of the maxilla, absent stylopodial bone, truncated zeugopods, ectopic cartilage tissue formation	29
*Fgf8*^*H/−*^	LOF (hypomorphic)		Micrognathia, bony cleft palate, temporomandibular joint fusion, absent tympanic ring, cardiovascular malformations, cerebellum hypoplasia, olfactory bulbs hypoplasia	32
			Lung hypoplasia	90
*Fgf8;Nes*^*Cre*^	LOF (biallelic)		First arch syndromes, agnathia	33
*Fgf8*^*Δ/Neo*^	LOF (hypomorphic)		Abnormal pharyngeal arch development	35
			Bilateral syngnathia	37
*Fgf8*^*Neo/Neo*^	LOF (hypomorphic)		Absent coronoid process, truncated and dysmorphic lower jaws	37
			Corpus callosum hypoplasia	155
*Osr2*^*Cre*^*;Rosa26R*^*Fgf8*^	GOF		Micrognathia, masseter regression, disrupted patterning and differentiation of masseter tendon	38
*Foxc1*^*−/−*^*;Fgf8*^*Null/+*^	LOF (monoallelic)	Foxc1	Absent jugal, absent squamosal bone, shortened frontonasal region, small frontal process of maxilla, abnormal dentary morphology, syngnathic jaw, abnormal bone palate fusion	39
*Fuz*^*−/−*^*;Fgf8*^*lacz/+*^	LOF (monoallelic)	Fuz	Skull defects amelioration	45
*Tfap2a*^*+/−*^*;Fgf8*^*+/−*^	LOF (monoallelic)	Tfap2a	Cleft palate amelioration	46
*Msx2*^*Cre*^*;Fgf8*^*flox/Δ2,3*^	LOF (biallelic)		Abnormal limb morphology	56
*Fgf8*^*+/dup*^	GOF		Shortened proximal limbs	66
*Fgf8*^*dup/del*^	GOF/LOF		Shortened proximal limbs	66
*Nkx2.5*^*Cre*^*;Fgf8*^*flox/−*^	LOF (biallelic)		OFT/RV defect	84
*Fgf8*^*Neo/lacZ*^	LOF (hypomorphic)		OFT/RV defect	84
*MesP1*^*Cre*^*;Fgf8*^*C/−*^	LOF (biallelic)		Heart tubes hypoplasia, short or absent OFT, dilated ventricle and atrium	85
*Isl1*^*Cre*^*;Fgf8*^*C/−*^	LOF (biallelic)		RV hypoplasia, small pharyngeal arches	85
*Crkl*^*−/−*^*;Fgf8*^*+/−*^	LOF (monoallelic)	Crkl	Pharyngeal arch artery hypoplasia, aortic sac dilatation	95
*Tbx1*^*+/−*^*;Fgf8*^*+/−*^	LOF (monoallelic)	Tbx1	Aortic arch artery defect	97
*Fgf8*^*−/−*^	LOF (biallelic)		Renal hypoplasia, arrest at renal vesicle stage	99;101
*Fgf8*^*fl/−*^*;Fgf20*^*Cre/−*^	LOF (biallelic)	Fgf20	Bilateral kidney agenesis, kidney failure	104
*T*^*Cre*^*;Fgf8*^*flox/Δ2,3*^	LOF (biallelic)		Abnormal male reproductive tract development	105
*Rarb2*^*Cre*^*;Fgf8*^*flox/Δ2,3*^	LOF (biallelic)		Efferent ductules failure	106
*Fgf8*^*Δa/Δa*^	LOF (biallelic)		Growth retardation, postnatal lethality	125
*Fgf8*^*Δb/Δb*^	LOF (biallelic)		Absent midbrain, absent isthmus, absent cerebellum	125
*Emx1*^*Cre*^*;Fgf8*^*flox*^	LOF (biallelic)		Midline defect	129
*Fgf8*^*HOMO*^	LOF (hypomorphic)		Decreased GnRH neuron number	135
*Fgf8*^*HET*^	LOF (monoallelic)		Decreased GnRH neuron number	135
*Fgf8*^*+/Neo*^	LOF (hypomorphic)		Decreased vasopressin neuron number	136
			Elevated anxiety-like behavior	162
*Chd7*^*+/−*^*;Fgf8*^*+/−*^	LOF (monoallelic)		Decreased cerebellar size	151
*Fgf8*^*Null/Neo*^	LOF (biallelic)		Abnormal external ear morphology, absent squamosal bone, absent proximal processes of dentary, absent nasal bone, mandible hypoplasia	155

Abbreviations: GOF, gain of function; LOF, loss of function; PTA, Persistent truncus arteriosus; OFT, outflow tract; RV, right ventricle; GnRH, gonadotropin-releasing hormone.

### Nonsyndromic cleft lip and palate (NSCLP)

Nonsyndromic cleft lip and palate (NSCLP), a complex congenital defect influenced by genetic and environmental factors, has been associated with *FGF8* mutations. In a study by Riley BM et al, genome sequencing was employed to identify a de novo *FGF8* missense mutation (p.Asp73His) in a Lowa patient with bilateral cleft lip and palate. In silico predictions revealed that the p.Asp73His mutation could induce a conformational alteration in the FGF8 protein, destabilizing FGF8 N-terminal conformation, reducing the binding affinity, and resulting in a loss of function^41^ (Table 2). In a parallel study, Dabrowska J and colleagues identified a variant in *FGF8* (p.Glu200Gly) in one of 135 Polish patients with NSCLP. This heterozygous mutation was inherited from the patient's mother, who also had a bilateral cleft lip and palate.^42^ Although it is unknown whether the current missense mutation in *FGF8* leads to functional loss or activation, it suggests that rare coding variants in *FGF8* might contribute to the genetic architecture of orofacial clefts.

### Orofaciodigital syndromes

Orofaciodigital syndrome (OFD) is a rare ciliopathy characterized by deformities of the face, oral cavity, digits, and brain. This syndrome is caused by pathogenic variants in genes encoding the ciliogenesis and planar polarity effector (CPLANE) complex, such as Fuzzy (Fuz) and orofacial digital syndrome-1 (Ofd-1), leading to various skeletal developmental disorders and ciliary dysfunction.^43^ J M Tabler et al demonstrated that overactivity in FGF signaling, due to increased cranial *Fgf8* gene expression in *Fuz* and *Ofd1* knockout mice, leads to craniofacial defects. Reducing the *Fgf8* gene dose alleviates these defects in *Fuz* mutant mice. This research indicates that FGF dysfunction causes facial defects in mutant mice with ciliopathy, establishing an etiological link between ciliopathies and FGF-hyperactivation syndromes through FGF8.^44^^,^^45^

Moreover, in humans, *TFAP2A* haploinsufficiency causes Branchio-oculo-facial syndrome, including multiple craniofacial abnormalities, skin defects, eye defects and hearing problems. Loss of function of *Tfap2a* in mice results in major defects in development of the head, with fully penetrant bilateral facial clefting with concomitant perinatal lethality, as well as, the bilateral clefting phenotype can be ameliorated by reducing the gene dosage in *Fgf8* heterozygote mice.^46^ *Fox3* mutants, exhibiting severe craniofacial skeleton defects, including the absence of all but the most distal tip of the mandible, show a correlation with a delay in *Fgf8* expression in branchial arch ectoderm.^47^ These observations indicate that *Fgf8* dosage plays a critical role in regional patterning and craniofacial morphogenesis, contributing to various hereditary craniofacial disorders.

## FGF8 in limb development and defect

### FGF8 in limb development

Vertebrate limbs originate from the lateral plate mesoderm (LPM) and surface ectoderm. The limb buds undergo progressive thickening, developing two successive rings, proximal and distal, each undergoes division into three distinct segments. The upper limb bud gives rise to the upper arm, forearm, and hand, while the lower limb bud differentiates into the thigh, leg, and foot. The position specification of limb formation in LPM involves the coordination of *Hox* and *Tbx* genes.^48^^,^^49^ Following this, LPM cells proliferate and expand outward, with the protrusion of LPM stromal cells inducing the organization of epiblast cells into the apical ectodermal ridge (AER).^50^^,^^51^

FGF8 signaling in AER is essential for normal limb bud development, initiating with forelimb buds emergence at E9.5 and hindlimb buds at E10 in mice.^52^^,^^53^ *Fgf8* expression is localized in the distal regions of developing mouse limb buds, restricted to AER.^54^ Fully formed AER exhibits detectable *Fgf8* expression along its entire length, persisting until regression occurs.^55^ FGF8 maintains epithelial-mesenchymal signaling, ensuring the timely generation of correct mesenchymal progenitor populations. Reduced *Fgf8* activity in AER leads to diminished limb bud size, delayed *Shh* expression, dysregulated *Fgf4* expression, and hypoplasia or aplasia of specific skeletal elements.^56^^,^^57^ *Fgf4*, *Fgf8*, *Fgf9*, and *Fgf17* are found to have AER-specific expression in the mouse limb bud.^58^^,^^59^ While deficiencies with *Fgf4*, *Fgf9*, and *Fgf17* display a normal skeletal pattern, the deletion of *Fgf8* alone results in a phenotypic signature characterized by shorter bones with smaller limb buds.^56^

Interdigital cell death (ICD), integral to interdigital separation in species such as chicken and mouse, is triggered by the downregulation of *Fgf8* expression in the ectoderm. *Fgf8* modulates retinoic acid (RA) levels by reducing *Raldh2* expression and enhancing *Cyp26b1* expression. Consequently, RA leads to decreased *Fgfr1* expression and *Erk1/2* phosphorylation. The coordinated action of *Fgf8* and RA regulates ICD, significantly contributing to the development of hands and feet.^60^^,^^61^

In chick embryo limb development, the *Fgf8* signal is specifically localized to the muscle and tendon boundary regions from E8 to E10, suggesting its involvement in mediating muscle–tendon interactions.^62^ Notably, in chicken embryos, *Fgfr4* exhibits abundant expression in most myoblasts within skeletal muscles. Inhibition of FGFR4 signal reduces limb muscle and impedes muscle progenitor differentiation. Conversely, *Fgf8* overexpression promotes *Fgfr4* expression and enhances muscle differentiation.^63^ Genetic studies in zebrafish embryos have identified *fgf8* as a crucial regulator of *scube3*-mediated fast muscle differentiation.^64^ *Lbx1* is a key regulator of limb myoblasts migration, and undergoes phosphorylation facilitated by FGF8 and ERK-mediated signaling, guiding the lateral migration of myoblasts toward the distal limb buds.^65^

### FGF8 dysregulation and limb defects

Inactivation of *Fgf8* in early mice limb ectoderm results in a notable reduction in limb bud size, disrupted proximal-distal patterning, increased apoptosis of limb bud cells, and consequent aplasia or hypoplasia of specific skeletal elements.^56^ De novo heterozygous duplications of *FGF8* have been identified in individuals with bilateral femoral duplications from unrelated families. Through an analysis of local chromatin architecture in individual cells and CRISPR-Cas9 editing mice, these duplications have been linked to ectopic chromatin contacts and increased *FGF8* expression. Transgenic mice with *Fgf8* heterozygous tandem duplication display proximal limb shortening, resembling the human phenotype. Capture Hi-C experiments conducted in *Fgf8*^*+/dup*^ limb buds further revealed a gain of chromatin interactions with the duplicated region, corroborating *Fgf8* overexpression. This suggests that position effects alter *FGF8* expression and contributed to the observed phenotype.^66^

### Split Hand/Foot Malformation (SHFM)

Split Hand/Foot Malformation (SHFM) is a congenital disorder characterized by variable defects of the central rays of the hands and/or feet, exhibiting substantial phenotypic variability within and across families, including polydactyly, syndactyly and monodactyly.^67^ SHFM is intricately linked to AER deficiency during limb bud development. AER-specific deletion of *Fgf8* in mice displays a normal AER, suggesting abnormalities in *Fgf8* expression alone may not be the sole cause of SHFM. However, mutations in SHFM-related genes within AER such as *Tp63*, *Wnt10b*, *Dlx5*, *Dlx6*, and *Fgfr1*, induce *Fgf8* dysregulation, disrupting the WNT-BMP-FGF pathways in AER. This leads to misexpression of AER genes, failure in AER stratification, and ultimately development of SHFM.^68^^,^^69^ Moreover, tandem duplication at the *Lbx1/Fgf8* locus triggers the ectopic activation of *Lbx1* and *Btrc* genes within AER, contributing to manifestation of SHFM3.^70^ SHFM3 was reported in several patients with 10q24 tandem heterozygous duplications, with varying degrees of severity.^71^^,^^72^

### Other limbs abnormalities

Exposure to dexamethasone (Dex) during chicken embryo development impacts the FGF signaling pathway by reducing *Fgf8* expression.^73^ Maternal Dex exposure induces alterations in RA signaling mediated by FGF-ERK signaling, ultimately affecting limb development in offspring.^74^ Additionally, gestational ethanol exposure reduces *Fgf8* expression in the AER and *Shh* expression in the polarized active zone, disrupts the AER/ZPA positive feedback loop, and induces postaxial malformations. Higher ethanol dosages cause preaxial deformities, indicating that *Fgf8* serves as a pivotal target of ethanol in the development of limb defects.^75^ Mild hands abnormalities can be seen in patients with fetal alcohol spectrum disorders.^76^

### Muscular dystrophy

Limb girdle muscular dystrophies (LGMDs) are a heterogeneous group of an autosomal recessive limb-girdle muscular dystrophy with relative sparing of heart and bulbar muscles, except for some subtypes. In humans, *TRAPPC11* homozygous truncating and deletion have been reported in various families showing LGMD or myopathy with movement disorder.^77^^,^^78^ In zebrafish, depletion of *trappc11* suppresses FGF8, and leads to abnormal activation of Notch signaling, triggering epithelial-mesenchymal transition and fibrotic changes within the skeletal muscle. These alterations manifest as myopathic phenotypes, including disrupted muscle organization and myofibrosis development.^79^

## FGF8 in cardiopulmonary development and disease

### FGF8 in cardiopulmonary development

#### Cardiovascular development

During embryonic development, the cardiovascular system originates from at least four different cell lineages: the first heart field (FHF), the second heart field (SHF), the cardiac neural crest and proepicardial organs.^80^^,^^81^ The vertebrate heart forms from FHF and SHF cardiac progenitors located in the LPM.^82^ The FHF differentiates into the early cardiac tubes, forming the part of the double atrium and left ventricle, while the SHF contributes to the formation of the right ventricle (RV) and the cardiac outflow tract (OFT).^80^^,^^83^ FGF8 plays a crucial role in the development of SHF, as evidenced by *Fgf8*-deficiency mice displaying reduced cell proliferation and abnormal cell death in the SHF pharyngeal endoderm and splanchnic mesoderm, resulting in the OFT/RV defects. Subsequent analysis identified *Fgf8* as the primary FGF ligand driving SHF development, with activating phosphorylated ERK and PEA3.^84^

During the SHF and early somite stages, *Fgf8* exhibits widespread expression in both the SHF mesoderm and pharyngeal endoderm. *Dll4*-mediated Notch signaling sustains *Fgf8* expression, essential for SHF progenitor cell proliferation through transcriptional regulation.^84^ Autocrine Fgf8 signaling derived from SHF mesoderm is essential for the formation of primary heart tube, RV and OFT myocardium. The absence of *Fgf8* in the mesoderm disrupts the development of RV/OFT myocardium, impacting the rotation/alignment of the OFT, while Fgf8 originating from the pharyngeal endoderm regulates OFT septation.^85^

Furthermore, FGF8 serves as chemokinetic and chemotactic signals for cardiac neural crest migration via Fgfr1 and Fgfr3, involving MAPK/ERK intracellular signaling.^86^ As a downstream secreted factor of *Tbx1*, *Fgf8* represses *Sema3c* expression in cardiac neural crest cells, inhibiting migration and causing abnormal chick pharyngeal arch development by activating the ERK1/2 signaling pathway.^87^

#### Lung development

*Fgf8* involves in lung development, crucial for alveolar formation, as evident from its expression detected in the lungs of E12.5 mice and adult rats.^88^^,^^89^ In the developing mouse lungs, *Fgf8* expression persists from E12.5 to postnatal day 1 (P1), with a notable increase at P5. Analyses of isolated epithelial and mesenchymal cells from E15.5 mouse lungs revealed a relative enrichment of *Fgf8* transcripts in the epithelial cells, definitively establishing that red blood cells or other circulating cells are not the sources of *Fgf8* transcripts in the lung. In *Fgf8*^*H/-*^ mutant mice, there is an observation of hyperplastic lungs with septal thickening at E18.5. The *Fgf8* deficient hypomorphs (*Fgf8*^*H/−*^, animals bearing a hypomorphic allele and a null allele of Fgf8) lead to a hyperproliferative lung phenotype attributed to mesenchymal excess proliferation. Transcriptomic analysis corroborates that reduced FGF8 signaling causes dysfunction in pathways regulating proliferation, intercellular and cell-extracellular matrix interactions.^90^

*Fgf8* and *Fgf18* emerge as the most closely related ligands expressed in lung epithelium. However, it is crucial to emphasize that the phenotypic manifestations of *Fgf18* mutants significantly deviate from those observed in *Fgf8* mutants in mice. *Fgf18* null mutants present with hypoplastic lungs characterized by diminished cell proliferation and preserved epithelial differentiation. In contrast, Fgf8 hypomorphic mutants exhibit a hyperplastic phenotype marked by overproliferation in both distal epithelial and mesenchymal compartments, accompanied by disrupted distal epithelial differentiation. This disparity underscores the distinct roles of Fgf8 in supporting fetal lung development. Insufficient *Fgf8* function results in pronounced overproliferation spanning both epithelial and mesenchymal compartments from E16.5 to E18.5, leading to subsequent disruptions in epithelial differentiation and abnormal septal and vascular remodeling.^90^^,^^91^

## FGF8 dysregulation results in cardiovascular pathogenesis

### Congenital heart disease (CHD) and OFT pathogenesis

Congenital heart disease (CHD) is the most prevalent, life-threatening congenital malformation, with OFT recognized as primary hotspot for CHD pathogenesis. Previous studies have revealed at least four mutually nonexclusive roles of Fgf8 in contributing to OFT defects. Firstly, the loss of *Fgf8* during gastrulation impedes the formation of the FHF from cardiac precursor cells.^84^ Secondly, *Fgf8* deficiency disrupts proper cardiac asymmetric development, thereby influencing OFT development.^92^^,^^93^ Thirdly, abnormal *Fgf8* expression leads to cardiac OFT septal defects by causing defects in cardiac neural crest cells.^94^ Lastly, starting as early stage E7.5, *Fgf8* becomes expressed in the mesoderm, potentially playing a direct role in maintaining or specifying the SHF. Diminished SHF and subsequent OFT shortening can result in OFT septal defects.^55^

### Persistent truncus arteriosus (PTA)

Persistent truncus arteriosus (PTA) is a rare congenital heart defect characterized by a single arterial trunk arising from the heart, supplying blood to the pulmonary, coronary, and systemic circulations. The etiology of PTA remains unclear but is hypothesized to result from the failure of the embryonic truncus arteriosus to properly divide into the aorta and the pulmonary artery, leading to a mixture of oxygenated and deoxygenated blood. There is a notable association between truncus arteriosus and 22q11.2 deletion syndrome. Overexpression of *Fgf8* in the cardiac neural crest hinders the formation of aorticopulmonary septum by inhibiting endothelial differentiation and stimulating the proliferation of endocardial pad cells. *Fgf8* gain-of-function mutations impair epithelial-mesenchymal transition by disrupting FGF and BMP signaling, causing a novel type of PTA characterized by hyperplastic endocardial cushion.^94^

### Chromosome 22q11 deletion syndrome

The deletion of chromosome 22q11.21, the most common mircodeletion syndrome in humans, disrupts pharyngeal and cardiac development, resulting in malformations of the heart, outflow tract, and vasculature. *Fgf8* participates in the pathogenesis of 22q11 deletion syndrome by inducing *Fgfr1* and *Fgfr2* tyrosine phosphorylation, which promotes their binding to *Crkl*.^95^ *Tbx1*, one of the main genes responsible for the etiology of the syndrome, is required for efficient incorporation of cardiac progenitors of the second heart field (SHF) into the heart.^96^ *Tbx1* and *Fgf8* have been identified as genetic interactors during aortic arch development in crossed *Tbx1* and *Fgf8* mutants. This interaction significantly impacts the penetrance of cardiovascular defects in individuals with chromosome 22q11 deletions involving TBX1.^97^ *TBX1* is required to sustain ECM-intracellular signaling and that integrin-focal adhesion integrity in cardiac outflow tract development.^98^ These findings suggest that *TBX1* and *FGF8* co-regulation of the ECM-cell interaction is a critical event of early heart morphogenesis and a candidate pathway in the etiology of CHDs.

## FGF8 in urogenital system development and defect

### FGF8 in urogenital system development

#### Nephrogenesis and Fgf8 role

*Fgf8* is essential for cell survival during various stages of nephrogenesis and regulates gene expression in the nascent nephrons. The initiation of *Fgf8* expression occurs in the kidney primordium around the emerging ureteric bud at E12, persisting until E18.5 in mice. Throughout nephrons mature, the FGF8 signal becomes restricted to a specific subset of the cell population within the epithelium and renal tubular progenitors in S-shaped bodies.^99^ This pattern of the proportion of *FGF8*-positive cells in kidney metanephric cells is consistent with observations in the human fetal gene expression (Fig. 1E).

Renal vesicle-secreted *Fgf8* binds to *Fgfr1*, providing essential support for the survival of nephron progenitors.^100^ *Fgf8* collaborates with *Wnt4* to induce *Lim1* expression, thereby promoting metanephric mesenchyme survival and tubulogenesis.^101^ Notably, *Fgfrl1* knockout mice, lacking *Fgf8* expression in metanephric mesenchyme, display the absence of metanephric kidneys, indicating the crucial roles of *Fgfrl1* and *Fgf8* in nephrons formation.^102^ Early deletion of *Fgf8* impedes the condensation of nephron progenitor to the ureteral bud, resulting in delayed nephron formation and subsequent postnatal mortality.^100^^,^^101^ This suggests the robust chemotactic exerted by *Fgf8*.^103^ *Fgf8* drives nephrogenesis independently of *Spry1*, *Fgf9* and *Fgf20*. In mice, simultaneous deletion of both *Fgf8* and *Fgf20* leads to renal agenesis, impaired proliferation of nephron progenitors, and cell death. Removing a single *Fgf8* copy counteracts the effects of one copy of *Spry1*, effectively rescuing kidney agenesis caused by the deletion of *Fgf9* and *Fgf20*.^104^

### FGF8 involvement in male reproductive tract development

FGF8 plays a crucial role in orchestrating the development of the male reproductive tract by regulating the epididymis, vas deferens and efferent ductules. This regulation occurs through the growth and maintenance of the cranial region of the Wolffian duct along with the mesonephric tubules providing progenitors. In *T-Cre;Fgf8*^*flox/Δ2,3*^ mice lacking *Fgf8* expression in the mesoderm, there is evidence of incomplete cranial mesonephric tubules at E10.5 and deficient epididymis and efferent ductules at E18.5, resulting in incomplete development of the male reproductive tract. Recently, research indicated that FGF8 signaling within the kidney mesenchyme supports nephron progenitor survival by inactivating BAX/BAK-mediated apoptosis during mbryonic kidney development.^105^ *Rarb2-Cre;Fgf8*^*flox/Δ2,3*^ mutants, which lose *Fgf8* expression in mesonephric tubules but not somites, manage to develop the epididymis and vas deferens but fail to form efferent ductules. This highlights the indispensable role of *Fgf8* expression in the mesonephric tubules for the establishment of efferent ductules.^106^

### FGF8 dysregulation leads to renal disease

#### Polycystic kidney formation

In metanephric development, the improper connection between distal tubules and collecting duct epithelium leads to urine accumulation within the renal tubules, potentially resulting in cysts formation and polycystic kidney. *Pax2* haploinsufficiency in humans causes Papillorenal syndrome, characterized by various renal malformations and cysts. Kispert and colleagues observed that the misexpression of *Fgf8* driven by the *Pax-2* upstream region in transgenic mice resulted in enlarged kidneys with larger cysts.^107^ Although there is no genetic evidence of *FGF8* mutations in human Papillorenal syndrome, these findings suggest that ectopic expression of *FGF8* in the Wolffian duct and its derivatives contributes to polycystic kidney disease.

#### VATER/VACTERL syndromes

The VATER/VACTERL syndromes present with features like renal malformations, limb defects, and cardiac defects, predominantly manifest as renal phenotypes. Zeidler and coworkers identified two *FGF8* heterozygous missense variants (p.Gly29_Arg34dup, p.Pro26Leu) in two different patients by conducting a targeted *FGF8* sequencing on 78 VATER/VACTERL and VATER/VACTERL-like individuals, and both patients presented with bilateral cryptorchidism. This finding suggests a potential role of *FGF8* in renal malformations associated with VATER/VACTERL syndrome, but it has not yet been studied in greater depth.^108^^,^^109^

### FGF8 in neurodevelopment and disorder

#### FGF8 in neurodevelopment

Numerous studies highlight the important role of FGF8 in brain development, particularly its necessity for the proper formation of the forebrain (telencephalon and diencephalon), midbrain, and hindbrain.^12^^,^^110^ It is further supported by the substantial expression and proportion of *FGF8* observed in the fetal development profile. The precision in both the localization and dosage of *FGF8* proves to be a critical determinant in initiating downstream signaling factors, ultimately contributing to the establishment of the pattern and orientation of various brain regions.^111^, ^112^, ^113^

### FGF8 in forebrain

The precise orchestration of *FGF8* expression is vital for ensuring the viability of cells within the forebrain. This regulatory role is executed through the FOXG1 pathway in a dose-dependent manner, as elucidated by studies.^114^ Notably, *Fgf8*^*neo/null*^ mice exhibit reduced cellularity post E15.5, indicating the trophic effects of FGF8 signaling on the retromamillary area, extending to all cells migrating tangentially from the ventral premamillary nucleus and subthalamic nucleus. Furthermore, FGF8 plays a dual role in regionalization and differentiation of the forebrain. The anterior neural spine (ANR) acts as an organizing center of the forebrain by generating FGF8 morphogen.^115^^,^^116^ Proper apoptosis in the ANR's specific subdomain is required to eliminate FGF8-producing region, ensuring proper forebrain development. Apoptosis-deficient mutants lead to the accumulation of undead cells, persistent *Fgf8* expression, and incomplete closure of the cranial neural tube, thereby impeding adequate brain ventricle expansion.^117^

Insights into the role of *Fgf8* in forebrain midline development come from the analysis of zebrafish mutants. The absence of *fgf8* activity leads to midline cells failing to adopt normal morphology, causing severe commissural axon pathway defects.^118^ Additionally, FGF8 is implicated in anterior-to-posterior (A/P) patterning.^117^ Acting as a diffusible morphogen alongside *Fgf17* and *Fgf18*, *Fgf8* imparts A/P locational information to the cortical primordium through in utero electroporation. Overexpression of anterior *Fgf8* induces a posterior shift in cortical areas. The parallels in cortical gene expression between *Emx2* mutants and *Fgf8* overexpressing mice suggest a mutually antagonistic involvement of *Fgf8* and *Emx2* in cortical region development.^119^, ^120^, ^121^

### FGF8 in midbrain and hindbrain

The development of the vertebrate midbrain and cerebellum is intricately governed by the isthmic organizer (IsO) center situated at the midbrain-hindbrain (MH) border. The distinct spatial–temporal expression patterns of *Fgf8* gene within the prospective isthmic domain strongly suggest FGF8's pivotal role in the formation and maintenance of the isthmus, involving crucial events in the MH domain.^111^^,^^112^^,^^122^^,^^123^ Gain-of-function studies have revealed that mouse *Fgf8a* and *Fgf8b* differ in activities due to distinct binding affinity to the FGF receptor.^124^ Deletion of *Fgf8b*-containing spliceforms, similar to loss of *Fgf8*, results in hypoplasia in midbrain, isthmus, and cerebellum. Intriguingly, mice lacking *Fgf8a* exhibit no obvious abnormalities, indicating the indispensability of *Fgf8b* spliceforms for FGF8 function during midbrain and cerebellum development.^125^

### FGF8 in ganglion neurons

FGF8 modulates the differentiation of the medial and caudal ganglionic eminence.^126^ Fgf8 regulates cell fate by promoting astrocyte differentiation and inhibiting neurogenesis.^127^^,^^128^ Differentiation of radial glia into astroglia, crucial in interhemispheric remodeling, is initiated by FGF8 signaling to downstream Nfi transcription factors.^129^ The migration and proliferation of oligodendrocyte progenitor cells are activated by FGF8 in vitro.^130^ Both FGF8a and FGF8b promote the outgrowth of spiral ganglion neurons neurites in vitro, mediated by FGF receptors and the activation of IκBα-mediated NFκB signaling, which is associated with the development and maintenance of language, learning, communication and social interaction.^131^^,^^132^

In summary, FGF8 stands as a key orchestrator of the intricate processes governing brain development. It exerts its influence with precision and specificity, ensuring the proper formation of crucial brain regions during fetal development.

### FGF8 dysregulation causes neurodevelopmental defects

#### Kallmann syndrome and hypothalamo-pituitary dysfunction

Kallmann syndrome (KS) is a genetically heterogeneous neurological disorder, characterized by hypogonadotropic hypogonadism due to lack of the production of sex hormones. Abnormal Gonadotropin-releasing hormone (GnRH) system development is a hallmark of KS. GnRH neurons, originating from the medial olfactory placode (OP), are crucial for reproduction.^133^^,^^134^ FGF8 signaling is required for GnRH neurons emergence from the early stage of embryonic OP. The intricate development and differentiation of GnRH neurons exhibit a high sensitivity to FGF8-FGFR1 signaling, following a time- and dose-dependent manner,^135^^,^^136^ which orchestrated by epigenetic mechanisms involving DNA and histone-modifying proteins.^137^^,^^138^ Misexpression of *Fgf8* leads to diminished GnRH neurons, reduced GnRH peptide concentration, and impaired embryonic migration, potentially leading to KS.^5^^,^^135^^,^^139^^,^^140^ Moreover, two heterozygous nonsense variants in *FGF8* (p.Arg127∗ and p.Arg129∗) were identified in two unrelated families with variable expressivity, including patients with isolated hypogonadotropic hypogonadism, isolated olfactory abnormalities, KS, and asymptomatic individuals.^141^

Furthermore, Fgf8 modulates the proportions of the frontal cortex (FC) and olfactory bulb in the anterior telencephalon and regulates the formation of the FC subdivisions.^142^^,^^143^ Neonatal mice with *Fgf8*^*+/neo*^ exhibit a reduction in vasopressin neurons in the paraventricular nucleus (PVN) and experience a disrupted onset timing of neuropeptide expression in postnatal PVN neurons. These findings provide partial explanations for affective disorders characterized by hypothalamic-pituitary-adrenal axis hyperactivity, such as anxiety.^144^^,^^145^

#### Holoprosencephaly

Holoprosencephaly (HPE) is a midline brain malformation characterized by the degree of separation of the eye field and telencephalon into distinct left and right structures. It results from incomplete cleavage of the prosencephalon into hemispheres. It presents a high heterogeneity, both in clinics and genetics. The phenotypic spectrum of HPE includes mild craniofacial features and severe manifestations like microcephaly, cyclopia, and a proboscis. Dysfunction in FGF8 is associated with malformations in various brain regions.^144^ Dubourg C et al provide a diagnosis in approximately 24 % of 257 HPE patients by developing a targeted next-generation sequencing (NGS). They discover that a various heterozygous *FGF8* mutations, and demonstrate that *FGF8* is a major gene involved in HPE.^146^ Interestingly, McCabe MJ et al identified a novel homozygous *FGF8* mutation (c.566G > A, p.Arg189His) in a female patient of consanguineous parentage with semilobar HPE, characterized by partial separation of the hemispheres and ventricles posteriorly.^144^ Both parents were heterozygous and had a normal phenotype with no history of delayed puberty or subfertility and a reported normal sense of smell. The phenotype of HPE associated with *FGF8* alterations is variable, such as the same inherited nonsense mutation (p.Arg129∗, stop gain) was identified in two unrelated patients, with difference degree form. It supports that another event could be necessary to lead to severe HPE. The human *FGF8* gene is relatively intolerant to sequence variation (pLI = 0.93; ExAC), and it could be sensitive to dosage.^147^ So far, no homozygous loss-of-function mutations have been found in a child whose parents carry heterozygous *Fgf8* mutation and with HPE phenotype. Moreover, Hong et al reported a unique case that a female patient inherited a deleterious *FGFR1* allele and a loss-of-function allele in *FGF8* from the both parents in one HPE family.^148^ These observations strongly suggest that the anomalous FGF8-FGFR1 signaling pathway plays a major role in HPE.

#### CHARGE syndrome

CHARGE (coloboma, heart anomaly, choanal atresia, retardation of mental development, genital hypoplasia and ear abnormalities) Syndrome, an autosomal dominant disorder caused by *CHD7* haploinsufficiency, is characterized by a non-random clustering of complex congenital malformations. Approximately 35 % of patients present with cerebellar vermis hypoplasia. Neurological dysfunction is a common feature in these patients, often manifesting as developmental delay, motor incoordination, intellectual disability, and autistic features.^149^^,^^150^ The reduction of *Fgf8* expression has been observed in *Chd7*^*+/−*^ mouse embryos.^151^ While *Chd7*^*+/−*^ mice exhibit normal cerebellar vermis, *Fgf8*^*+/−*^ mice display evident cerebellar vermis hypoplasia. This suggests an interaction between *Fgf8* and *Chd7* during cerebellar development, indicating that decreased FGF signaling links to cerebellar vermis hypoplasia in CHARGE syndrome.^151^, ^152^, ^153^

#### Acrocallosal syndrome

The formation of corpus callosum requires astroglial-mediated interhemispheric midline remodeling. In the presence of FGF8, primary astrocytes isolated from the perinatal brains develop more branches. FGF8-FGFR3 signaling facilitates the damage repair process in vitro by altering the morphology of astrocytes, potentially controlling astrocyte reactivity under injury conditions.^154^ Transgenic mice with *Fgf8* deficiency exhibit failed corpus callosum formation, and perinatal deficiency of *Fgf8* was found to cause impaired midline astrocyte development, hindering the formation of the corpus callosum.^155^ This impairment may potentially contribute to abnormal brain development in offspring, potentially leading to neurodevelopmental disorders. Acrocallosal syndrome (ACLS) is a rare autosomal recessive syndrome with corpus callosum agenesis, facial dysmorphism, post-axial polydactyly of the hands and pre-axial polydactyly of the feet caused by KIF7. In humans, increased FGF8 signaling is associated with corpus callosum abnormalities in KIF7-related ACLS, another ciliopathy.^156^

### FGF8 in neurotransmission and psychiatric disorders

The MH border generates a variety of neuronal populations including the dorsal raphe (DR) nucleus. Serotonergic neurons, located in DR, regulate stress-related behaviors and are involved in neuropsychiatric disorders such as anxiety and depression.^157^^,^^158^ Simultaneously, the localization of DA neurons along the anterior-posterior and dorso–ventral axes is determined by the integration of SHH and FGF8, which are necessary and sufficient conditions for inducing DA neurons. By SHH and FGF8 acting, DA progenitors “commit” to produce DA neurons and subsequently undergo “determination”, which requires the function of selectively activated transcription factors, *Nurr1* and *Ptx3*. These “committed” and “det ermined” DA neurons express key genes involved in DA neurotransmission at different periods of development. Among them, FGF8 protein, along with SHH, plays vital roles in fetal DA neuron induction both in vitro and in vivo. These conditions, marked by the integrated actions of FGF8 and SHH, are not only necessary but also sufficient for the induction of DA neurons. Subsequent commitment and determination of these neurons are facilitated by selectively activated transcription factors, specifically *Nurr1* and *Ptx3*.^159^^,^^160^ Moreover, FGF8 exerts influence over the axonal growth of midbrain DA neurons, primarily through the induction of sema3F.^161^ Fgf8 is a major regulator of serotonergic neuron development. *Fgf8*-deficient mice exhibit dysregulated serotonergic neuronal activation, serotonin metabolism, and elevated anxiety-like behavior.^162^ The spatiotemporal dynamic expression of *Fgf8* corresponds to distinct subpopulations of serotonergic neurons in the dorsal raphe nucleus, significantly contributing to functional heterogeneity.^157^

## Conclusions

**Figure 2:**
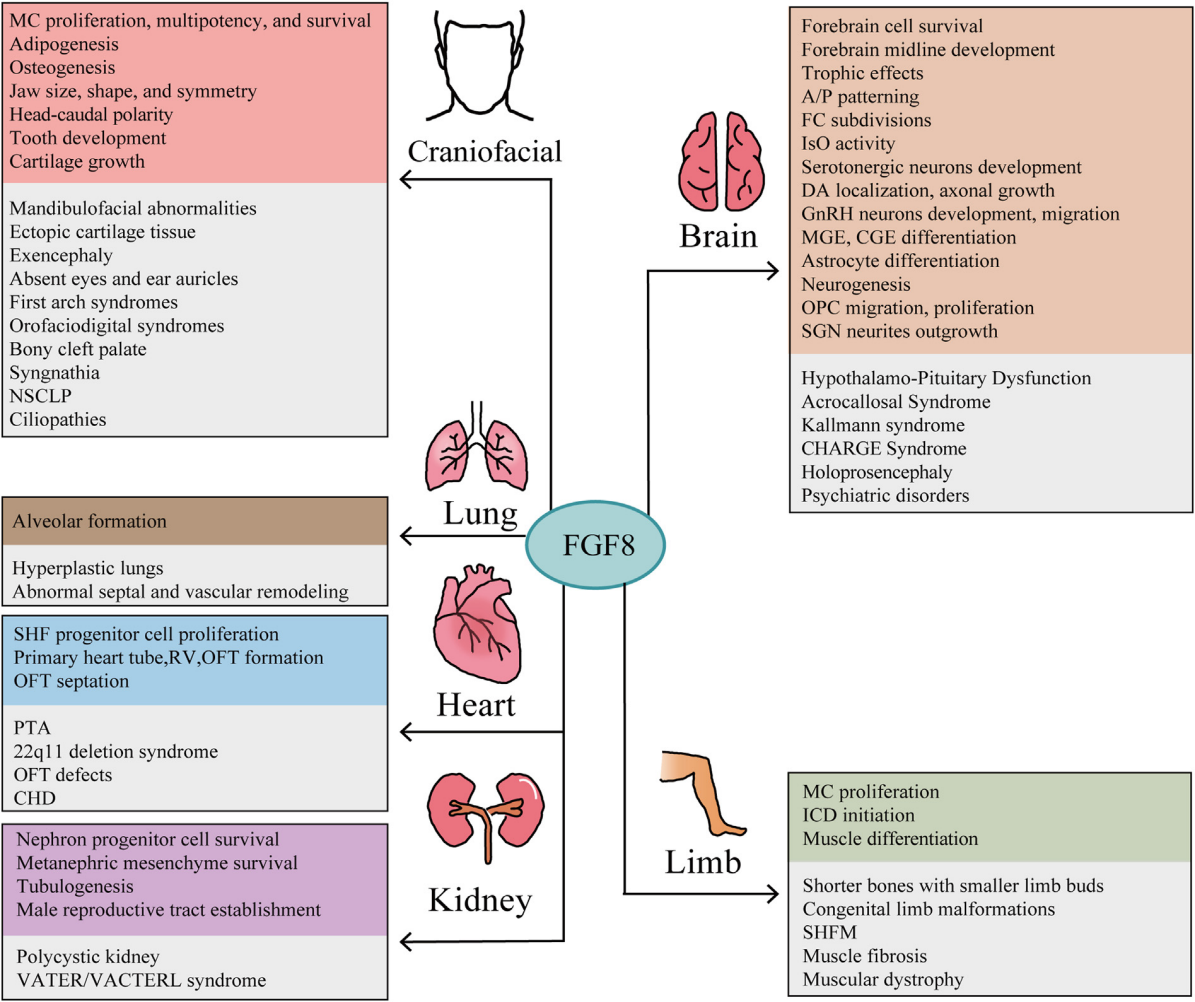
FGF8 assumes a pivotal role in the process of mammalian growth and development. FGF8 in craniofacial, limb, brain and internal organ development and development malformations (grey). MC: Mesenchymal cell; NCC: Neural crest cell; NSCLP: Nonsyndromic cleft lip and palate; IsO: Isthmic organizer; FC: Frontal cortex; A/P: Anterior to posterior; DA: Dopaminergic neurons; MGE: Medial ganglionic eminence; CGE: Caudal ganglionic eminence; OPC: Oligodendrocyte progenitor cell; SGN: Spiral ganglion neurons; GnRH: Gonadotropin-releasing hormone; ICD: Interdigital cell death; SHFM: Split hand/foot malformation; PTA: Persistent truncus arteriosus; SHF: Second heart field; OFT: Outflow tract; RV: Right ventricle.

Considerable evidence supports the essential role of precise spatiotemporal expression patterns and dosage of FGF8 in proper embryonic development. This growth factor plays crucial roles in various aspects of organogenesis, including craniofacial, limb, internal organ, and neural development. Disturbances in FGF8 levels during fetal development can result in organ malformations and subsequent multi-organ hypoplasia (Fig. 2). The advent of advanced techniques, such as high-throughput sequencing, enables comprehensive mapping of the entire transcriptome and regulatory profile of FGF8 during pregnancy and early development. Such advancements hold promise for unraveling the intricate mechanisms underlying FGF8 function. This knowledge can contribute to preventing developmental disorders in offspring, mitigating the risks associated with dysgenesis diseases, and easing the burden on both healthcare professionals and families.

## Funding

This study was supported by the 10.13039/501100001809National Natural Science Foundation of China (No. 81701350, 31671252); Health Technology Plan of Zhejiang Province (No. 2023RC205); The Natural Science Foundation of Zhejiang Province (No. LQ22C120002).

## CRediT authorship contribution statement

**Huamin Yin:** Writing – original draft, Visualization, Formal analysis, Data curation. **Lian Duan:** Writing – review & editing, Resources, Funding acquisition, Conceptualization. **Zhendong Wang:** Writing – review & editing, Supervision, Project administration. **Li Liu:** Writing – review & editing, Funding acquisition. **Jingling Shen:** Writing – review & editing, Supervision, Funding acquisition.

## Conflict of interests

The authors declare that there is no conflict of interests.
